# Comparative efficacy and safety of escitalopram, desvenlafaxine, and vortioxetine in the acute treatment of anxious depression: A randomized rater-blinded, 6-week clinical trial

**DOI:** 10.1192/j.eurpsy.2022.281

**Published:** 2022-09-01

**Authors:** K.-S. Oh, S.W. Jeon

**Affiliations:** 1 Kangbuk Samsung Hospital, Sungkyunkwan University School of Medicine, Department Of Psych, Seoul, Korea, Republic of; 2 Kangbuk Samsung Hospital, Sungkyunkwan University School of Medicine, Department Of Psychiatry, Seoul, Korea, Republic of

**Keywords:** escitalopram, desvenlafaxine, Depression, vortioxetine

## Abstract

**Introduction:**

This study is about the clinical uses of three antidepressants( escitalopram, desvenlafaxine, vortioxetine) in the treatment of anxioun depression.

**Objectives:**

The purpose of this study was to compare the efficacy and safety of escitalopram, desvenlafaxine, vortioxetine, and aripiprazole augmentation with escitalopram in the acute treatment of anxious depression.

**Methods:**

Patients (n=189) with DSM5 major depression and high levels of anxiety were evenly randomized to escitalopram, desvenlafaxine, vortioxetine, and aripiprazole augmentation with escitalopram in a six-week, randomized, rater-blinded, head to head comparative trial. Changes in overall depressive and anxiety symptoms were assessed.

**Results:**

Patients demonstrated similar baseline-to-endpoint improvement in HAMD and HAMA total scores. Patients also demonstrated similar response rate and remission rate in HAMD and HAMA. In analysis of individual HAMD and HAMA items, desvenlafaxine had greatly reduced scores for anxiety somatic (p=0.013), hypochondriasis (p=0.014), cardiovascular symptoms (p=0.005), respiratory symptoms (p=0.013) compared to escitalopram or vortioxetine. Each treatment were well tolerated with no significant differences.

**Conclusions:**

These results showed no significant differences in efficacy and tolerability of escitalopram, desvenlafaxine, vortioxetine, and aripiprazole augmentation with escitalopram in this subtype of patients with anxious depression during the acute phase treatment.

**Disclosure:**

No significant relationships.

